# Comparative Analysis of Tunicate vs. Plant-Based Cellulose in Chitosan Hydrogels for Bone Regeneration

**DOI:** 10.3390/gels11020102

**Published:** 2025-02-01

**Authors:** Laura Furlan, Annj Zamuner, Andrea Riccioni, Giacomo Sabbadin, Teresa Russo, Vito Gallicchio, Gabriella D’Auria, Lucia Falcigno, Lucia Manni, Loriano Ballarin, Elisabetta Schievano, Paola Brun, Monica Dettin

**Affiliations:** 1 Department of Industrial Engineering, University of Padova, 35131 Padova, Italy; laura.furlan@unipd.it (L.F.); annj.zamuner@unipd.it (A.Z.); 2 Department of Civil, Architectural and Environmental Engineering, University of Padova, 35131 Padova, Italy; 3 Department of Chemical Sciences, University of Padova, 35131 Padova, Italy; andrea.riccioni@polito.it (A.R.); elisabetta.schievano@unipd.it (E.S.); 4 Department of Biology, University of Padova, 35131 Padova, Italy; giacomo.sabbadin.1@studenti.unipd.it (G.S.); lucia.manni@unipd.it (L.M.); loriano.ballarin@unipd.it (L.B.); 5 Institute of Polymers, Composites and Biomaterials, National Research Council of Italy, 80125 Naples, Italy; teresa.russo@unina.it; 6 Department of Neurosciences, Reproductive and Odontostomatological Sciences, University of Naples Federico II, 80131 Naples, Italy; vito.gallicchio@unina.it; 7 Department of Pharmacy, University Federico II of Naples, 80131 Naples, Italy; gabriella.dauria@unina.it (G.D.); falcigno@unina.it (L.F.); 8 Department of Molecular Medicine, University of Padova, 35121 Padua, Italy; paola.brun.1@unipd.it

**Keywords:** plant-based cellulose, tunicate cellulose, chitosan, hydrogel, BMP-2, bone tissue engineering, structuring agent, design methods, mechanical measurement

## Abstract

A novel hydrogel scaffold for bone regeneration based on chitosan, selected for its biocompatibility, biodegradability, and antimicrobial properties, was covalently functionalized with a bioactive peptide from bone morphogenetic protein-2 (BMP-2) to guide osteoblast growth and proliferation. This study evaluates the impact of incorporating different concentrations (8, 16, or 24% *wt*/*wt*) of plant-based micro-fibrillated cellulose or tunicate nanocellulose to improve the mechanical and biological properties of peptide-grafted chitosan hydrogel matrices. While the mechanical properties of the matrices increase with increasing cellulose content, regardless of its source, the behavior of human osteoblasts used in biological tests discriminates between the two types of cellulose and shows better results (proliferation at 2 and 7 days, and mineralization) for the enrichment with tunicate cellulose.

## 1. Introduction

The increase in the incidence of bone defects caused by severe trauma, diseases, surgical resections, and radical tumor removal is a global health issue, extending to social and economic levels [1]. Approximately 45 billion dollars are spent annually to treat 15 million patients with bone disorders, 1.6 million of whom suffer from trauma-related fractures and 2 million have bone defects of osteoporotic origin. In particular, in the United States, 1.6 million patients undergo bone graft procedures each year, with a total cost of about 2.5 billion dollars, a figure that is expected to double by the end of the decade due to several factors, primarily the increase in individual life expectancy [2]. Bone repair is a complex process that involves the interaction of cells, molecules, and factors of a chemical, physical, and mechanical nature. Currently, the clinical solution for bone repair is represented by bone transplants from: (i) autologous material (autografts), which represents the present gold standard; (ii) a donor of the same species (allografts); (iii) a donor of a different species (xenografts); or, finally, (iv) synthetic materials [3,4]. The future perspective is represented by bone tissue engineering to create scaffolds that are capable of simulating the bone ECM [5]. The characteristics of an ideal scaffold for this application are biocompatibility, biodegradability with appropriate timing for the gradual replacement of the scaffold with bone tissue, the ability to promote cell adhesion/proliferation, osteogenesis, osteoinduction, osteoconduction, osteointegration, porosity, suitable mechanical properties, and reasonable cost [6,7].

**Figure 1:**
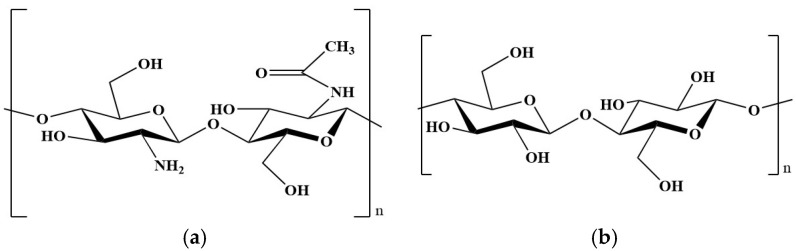
Chemical structure of: (**a**) chitosan (70% deacetylated chitin); (**b**) cellulose.

Chitosan (CS) is a natural polysaccharide, characterized by non-toxicity, biodegradability, biocompatibility, and antimicrobial properties; it is also generally recognized as safe by the Food and Drug Administration [8]. Moreover, chitosan-based bioactive materials have presented applications in cartilage, bone, skin, blood vessels, and corneal substitutes [9]. CS (Figure 1) derives from the partial deacetylation of chitin (poly-N-acetyl-glucosamine), the second most abundant polysaccharide in nature, which is obtained from crustaceans and insects, but also from fungi and bacteria [10,11]. Moreover, integrating biochemical signals into CS represents an attractive strategy for improving cell–biomaterial interactions.

To promote osteoblast proliferation and calcium deposition, a bone morphogenetic protein (BMP-2) mimicking peptide, called GBMP1α (sequence 48–69, PFPLADHLNSTNHAIVQTLVNS), was covalently linked to CS [7,12].

In this project, cellulose was added to peptide-grafted CS hydrogels to improve their mechanical properties.

Cellulose (poly-β-1,4-D-glucose, Figure 1) is the most abundant natural polymer and exhibits excellent biological properties, such as biocompatibility, non-cytotoxicity, water-retention capacity, and favorable mechanical properties like tensile strength and toughness, which can be modulated based on the polymerization degree, crystallinity, and chain orientation. Additionally, cellulose is widely available, environmentally sustainable, easily processable, and cost-effective [13,14]; native cellulose is not degradable by the human body due to the absence of enzymes that are capable of breaking the β-1,4-glycosidic bonds [4], but its nanometric form is considered degradable [15,16]. Recently, cellulose has been used to produce bioink for 3D bioprinting and porous scaffolds for different applications: from wound dressing to bone and cartilage tissue engineering, from neural to vascular tissue engineering [17].

Cellulose can be obtained from plants and certain types of algae, from bacteria, and from tunicates [14]. In plants, cellulose chains are arranged in layers as fibrils, held together by a matrix containing two other components, lignin and hemicelluloses, which are challenging to remove during the extraction process [18,19]. The production of bacterial nanocellulose utilizes sugar fermentation; the process can be meticulously controlled and optimized to achieve the desired material properties [4,20].

Finally, cellulose derived from tunicates is obtained from the animal’s outer covering tissue, known as the “tunic”. The plasma membrane of epidermal cells in tunicates contains various enzymatic complexes responsible for the synthesis of this highly pure cellulose [21,22]. Tunicate cellulose nanofibrils’ shape and size vary depending on the species; typically, their length ranges from 100 nm to more than 2 µm, while their width varies from 10 to 30 nm [14]. From 1 kg of non-dried tunicates about 10 g of cellulose can be obtained, depending on the species and the extraction method used; it is a low yield, but some species are invasive and easy to breed [22]. Tunicate-derived cellulose exhibits a high purity degree because it is free from other substances, such as lignin and hemicellulose. It is characterized by a high molecular weight (with a degree of polymerization ranging between 700 and 3500 units), a low polydispersity index, and a high crystallinity (approximately 95%), making it structurally and morphologically superior to cellulose obtained from other sources and well-suited for more advanced technical applications. Specifically, the crystallinity of cellulose derived from wood usually ranges from 40% to 60%, while cellulose from bacteria and tunicates shows a higher crystallinity ranging from 80% to 100% [14].

This project aims to evaluate the biological and mechanical properties of hydrogel scaffolds made up of CS, functionalized with the peptide GBMP1α, for the repair of bone defects when enriched with different amounts of cellulose derived from two different sources: plant and tunicate. The first is the commercial plant-based microfibrillated cellulose (P-MC) Exilva^®^ obtained from Scandinavian spruce wood; the second is tunicate nanofibrillated cellulose (T-NC) extracted from tunicates. The comparison between cellulose of different sources is important for application purposes and, to date, the articles presenting this comparison are very few.

Cellulose from tunicates is widely used in the biomedical field, such as in tissue engineering (myocardium, bone, and wound healing) [23]. Three main advantages of its use in bone tissue engineering are: the control of adhesion, growth, and direction of myogenic cells; no immunoreaction; and an osteoconductive effect [24].

If blends between cellulose and chitosan have been studied since 1990 [25], the main application in the tissue engineering field is essentially the formulation of patches for wound healing [26]. The principal examples of the cellulose/chitosan blend in bone tissue engineering concern three-component formulations, such as nanohydroxyapatite/chitosan/carboxymethyl cellulose or Ag nanoparticles/chitosan/carboxymethyl cellulose [27,28].

On the other hand, the literature does not report blends between tunicate nanocellulose and chitosan for the formulation of innovative biomaterials.

Although hydrogels suffer from mechanical limitations and cannot be the basis of load-bearing bone substitutes, they are particularly useful in regenerating non-load-bearing bone defects, as externally supported fracture fillers, or coating materials combined with other materials due to their advanced biological properties.

## 2. Results and Discussion

**Figure 2:**
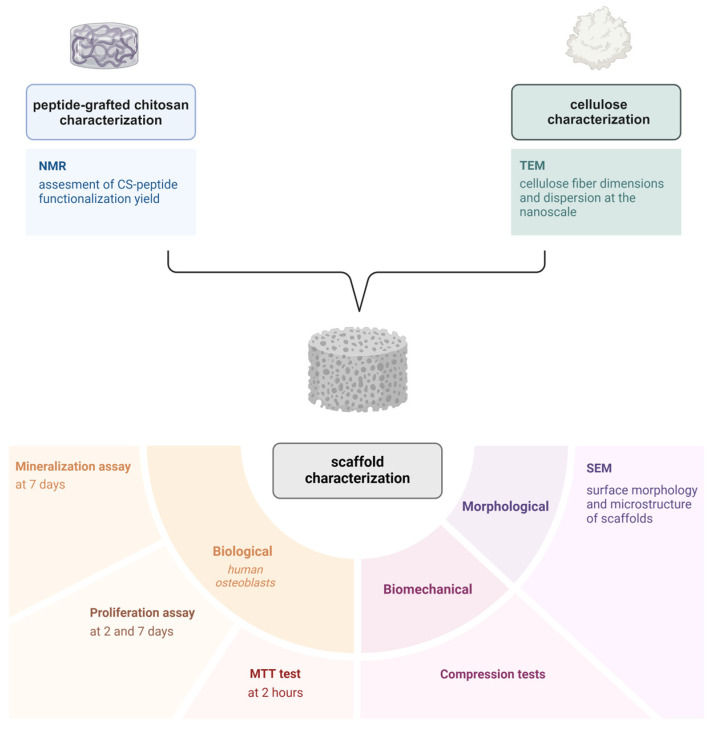
Scheme of all the tests performed for hydrogel matrices characterization.

To comprehensively evaluate the properties and performance of peptide-grafted chitosan scaffolds enriched with different types and concentrations (8, 16 or 24% *wt*/*wt*) of cellulose as a structuring agent, a series of chemical, morphological, mechanical, and biological tests were performed. The chemical characterization of peptide-grafted CS was carried out using nuclear magnetic resonance (NMR) to evaluate the functionalization degree of CS; plant-based microfibrillated cellulose (P-MC) and tunicate nanofibrillated cellulose (T-NC) components were morphologically analyzed via transmission electron microscopy (TEM) to assess their main structural properties. The scaffolds were morphologically characterized using scanning electron microscopy (SEM) to examine their structure and porosity. The hydrogels’ mechanical properties were evaluated through compression tests to determine their compressive modulus and maximum stress. Finally, biological characterization included osteoblast viability (at 2 h), proliferation (at 2 and 7 days), and mineralization (at 7 days) assays to evaluate bioactivity and potentiality for bone tissue engineering applications. All the tests performed on the samples are reported in Figure 2.

### 2.1. Chemical Characterization of Peptide-Grafted CS

#### Nuclear Magnetic Resonance (NMR)

A single source of chitosan was used to prepare two batches of CS-GBMP1α at different times. The degree of acetylation (DA) of chitosan was evaluated according to the literature and was found to be ~30–31% (Figure S1a) in agreement with our previous study. Therefore, approximately seven out of ten monosaccharides in the polymer are GlcN units available for peptide functionalization. The ^1^H NMR spectra of CS1 and CS2, i.e., of CS-GBMP1α used to obtain matrices enriched with the cellulose of vegetal or tunicate origin, are reported, respectively, in Figure S1b,c of the Supplementary Materials. The level of functionalization was assessed using the ratio R = I_H-pept_/I_H-CS_ between the integral corresponding to one proton of the peptide and the integral corresponding to one proton of CS. For both samples, the I_H-pept_ value was obtained by considering the integrals of specific signals, like those at 8.65 ppm due to the 2H of His^9^ and His^13^ of the peptide sequence. The I_H-CS_ was calculated using the integrals of the two CS regions (at 4.0–3.5 ppm and 3.18 ppm) reduced by the contribution of the overlapping peptide protons and dividing the result by 6, i.e., by the number of protons in each CS monosaccharide. In CS1, the peptide functionalization of CS was estimated to be ca. 25 ± 3%, i.e., ca. 25 monosaccharides out of 100 are linked to GBMP1α. The functionalization level of CS2 was found to be 8 ± 1%.

### 2.2. Morphological Characterization of Cellulose

#### Transmission Electron Microscopy (TEM)

**Figure 3:**
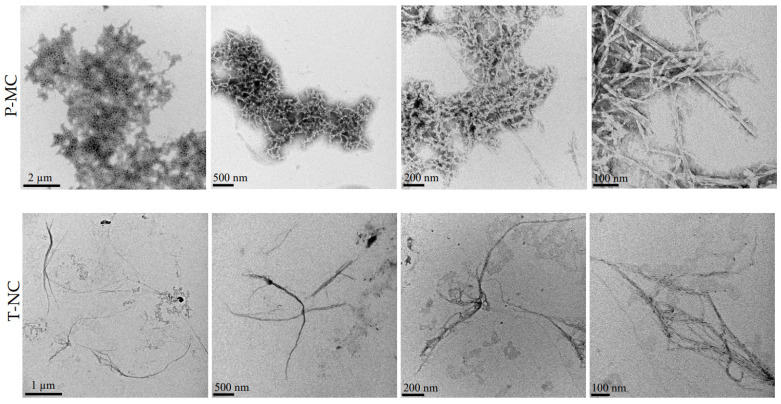
TEM analysis of P-MC and T-NC suspended in water.

The TEM analysis of P-MC in suspension revealed a structure composed of tightly intertwined and overlapping fibers. The fibers exhibited considerable variability in size, with smaller fibrils, predominantly in the nanometer range (Table 1). In contrast, T-NC showed a structure characterized by distinct fibers that were more evenly distributed within the suspension. These fibers were primarily of micrometric dimensions, with some fibers extending to only a few nanometers (Table 1). Overall, this analysis highlighted the different structures of P-MC and T-NC, which are represented in different magnifications in Figure 3. P-MC exhibited a more compact structure with generally smaller fibers that combine to form a dense network, whereas T-NC was characterized by longer well-separated fibers that form cord-like structures. The aspect ratio was calculated as the length/width of the fibers: T-NC fibers showed a higher value than P-MC fibers (261.1 vs. 13.9), as expected [23]; in particular, the value for T-NC extracted with our protocol (Section 4.2.3) results was higher than the aspect ratio reported in the literature [29].

### 2.3. Characterization of Hybrid Scaffolds

Briefly, to prepare a cellulose-enriched hydrogel matrix, CS and functionalized CS were dissolved in 0.2 M AcOH and mixed with the various specific amounts of cellulose suspended in a fixed volume of MilliQ water. Subsequently, matrices were frozen with liquid nitrogen, freeze-dried, washed with EtOH and 10× PBS, and finally freeze-dried. The matrices were re-hydrated with MilliQ water, left at −20 °C overnight, and lyophilized (cfr Section 4.2.4).

#### 2.3.1. Morphological Characterization

##### Scanning Electron Microscopy (SEM)

**Figure 4:**
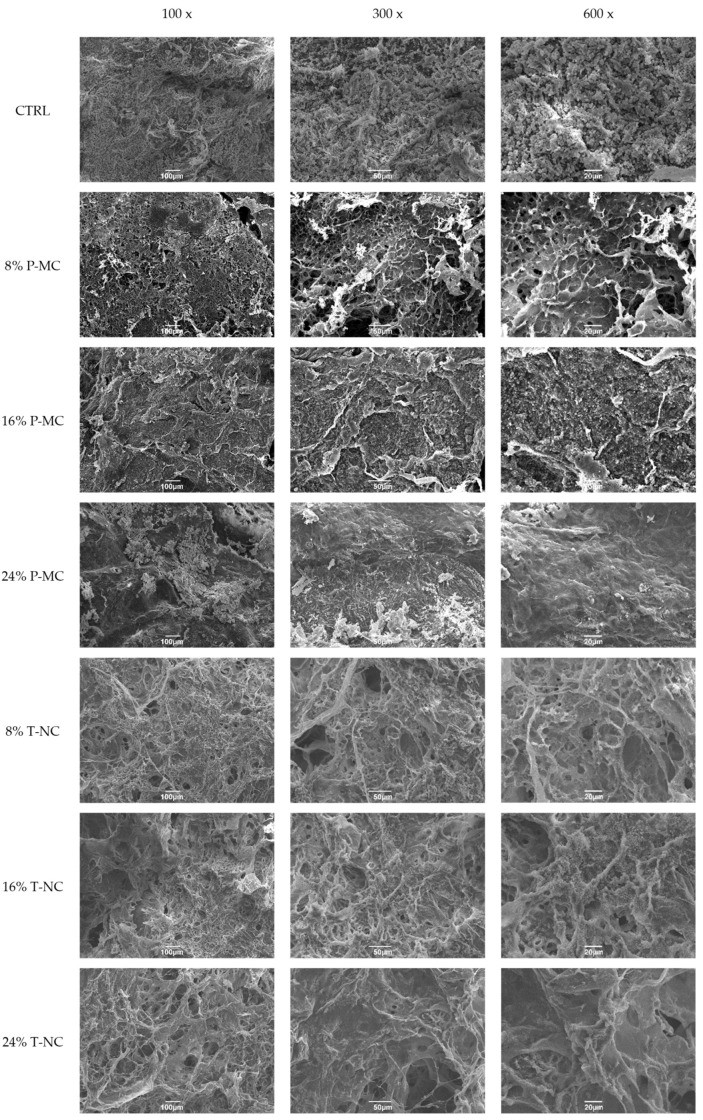
SEM analysis of the CTRL matrix and of those enriched with P-MC and T-NC at various concentrations (8%, 16%, 24%).

The control sample (CTRL), consisting of the freeze-dried hydrogel matrix made up of CS and CS functionalized with GBMP1α at a 1:1 ratio, exhibited a morphology characterized by spherical CS structures, consistent with the literature [30]. Upon the addition of 8% P-MC, the structure showed evident filamentous fibrils and the characteristic spherical CS structures were not easily detectable, resulting in a morphology similar to freeze-dried membranes made up of prevalently plant-based cellulose [31]. In the matrix containing 16% P-MC, the characteristic spherical CS structures reappeared and the filamentous fibril structure was still present—the two biomaterial components appeared more distinct; this matrix was less porous compared with 8% P-MC. In the matrix with 24% P-MC, there was a further noticeable reduction in porosity, along with a significant homogeneity in the spatial arrangement of the spherical CS structures and filamentous structures. Thus, the most P-MC-enriched matrix exhibited the most homogeneous and least porous structure, while the least-enriched matrix showed the most pronounced fibrillar structure and was the most porous matrix, consistent with the data presented in the literature [32]. In conclusion, compared with the control sample, samples containing 24% P-MC displayed a more uniform structure, with the filaments densely packed. All the SEM images for different magnifications are grouped in Figure 4.

Samples containing T-NC displayed a much more homogeneous structure with a filamentous arrangement, which became particularly pronounced at higher concentrations of T-NC. This suggests a significant impact of T-NC on the sample’s morphology, in particular, the increased number of filaments associated with higher concentrations of T-NC proportionally influenced the formation of the structure. In matrices enriched with 8 and 16% of T-NC, the fibrous structures showed surfaces decorated with typical spherical structures of CS, which presented smaller sizes than the pure CS ones. Differently, the morphology of matrices enriched with 24% T-NC showed a flake-like structure with the CS spherical elements less evident. The T-NC-containing samples demonstrated a more regular structure, which was densely populated with filaments compared with the control (Figure 4).

In conclusion, P-MC-enriched matrices exhibited heterogeneous morphology at different concentrations of cellulose; unexpectedly, the least concentrated condition showed the most fibrous and porous structure, which was similar to freeze-dried constructs made up of prevalently plant-based cellulose. On the other hand, T-NC-enriched matrices exhibited similar morphology at various concentrations and fluffier structures in which T-NC and CS were more homogeneously organized.

#### 2.3.2. Mechanical Measurements

##### Compression Tests

The compression tests performed on the first control group (CTRL1, hydrogel matrices made up of 50% CS + 50% CS1) and on all the groups consisting of hydrogel matrices enriched with P-MC revealed that the different kinds of analyzed structures exhibited qualitatively similar stress–strain curves, characterized by an initial linear region followed by a non-linear region up to the defined strain limit (40%) (Figure S2a). The compressive modulus (E) was calculated as the slope of the initial linear region of the stress–strain curve. The compressive modulus (E) and maximum stress (σ_max_) obtained for the matrices are reported as mean value ± standard deviation in Table 2. The results from the mechanical measurements indicated a progressive increase in both the maximum stress and compressive modulus values. In terms of maximum stress, the obtained findings were statistically significant (i.e., *p*-value of 0.005 for 8% P-MC enrichment, *p*-value of 0.0001 for 16% and 24% P-MC enrichment). Moreover, the maximum stress increased by 33% with the addition of 8% P-MC, by 53% with 16% P-MC, and by 87% with 24% P-MC. A statistically significant increase in the compressive modulus was also observed for the matrices with 16% P-MC (*p*-value of 0.01) and 24% P-MC (*p*-value of 0.0005). It doubled with the addition of 8% P-MC, increased by 2.5 times with 16% P-MC, and increased by 3.5 times with 24% P-MC. In conclusion, a progressive increase in the P-MC content significantly improved both the maximum stress and the compressive modulus of the structure, consistently with the data in the literature [33].

Furthermore, the results of the mechanical measurements in the second control group (CTRL2, hydrogel matrices made up of 50% CS + 50% CS2), and in all the groups consisting of hydrogel matrices enriched with T-NC, highlighted stress–strain curves for the different kinds of analyzed structures, which were similar to those found in the case of matrices enriched with P-MC (Figure S2b). Table 2 reports the compressive modulus (E) and maximum stress (σ_max_) for the second control group (CTRL2) and all the matrices enriched with T-NC. Also in this case, the results of the mechanical measurements generally showed a progressive increase in the values of the compressive modulus (E) and maximum stress (σ_max_). In particular, a significant difference was observed between the control matrix (CTRL2) and the matrices containing 24% T-NC. Specifically, the 24% T-NC enrichment provided the highest stress value and the highest compressive modulus. This suggested that an increase in T-NC content generally enhanced the maximum stress.

In conclusion, the matrices that were enriched with both types of celluloses presented improved the mechanical properties; however, there were no marked differences between the P-MC and T-NC enrichments on the matrices realized with this specific protocol and these percentual-low cellulose enrichments.

#### 2.3.3. Biological Characterization

##### Viability Assay

**Figure 5:**
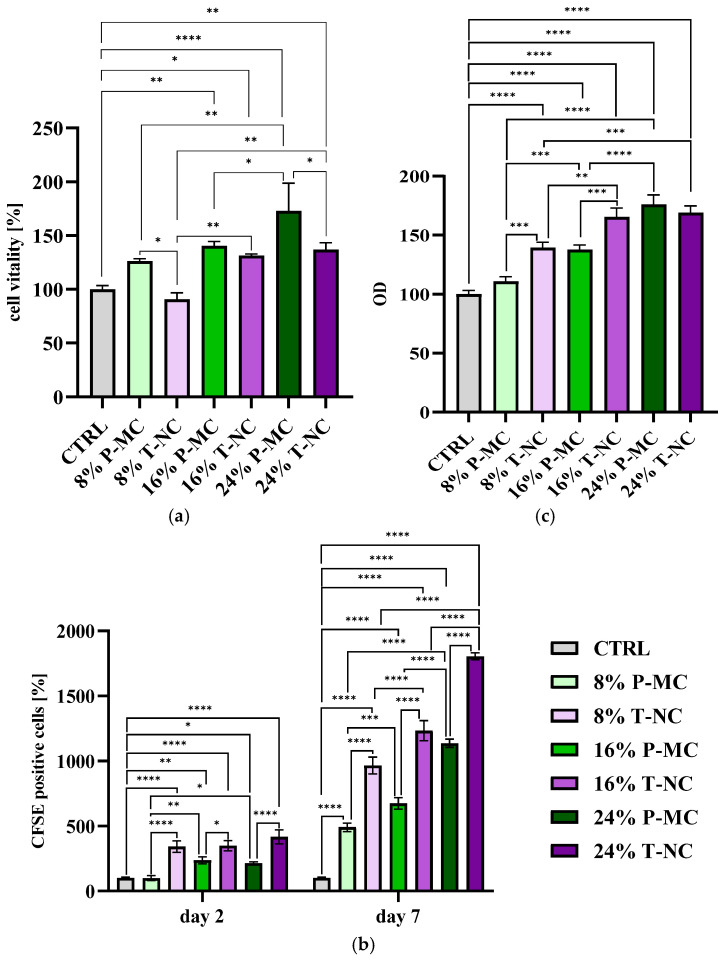
(**a**) MTT test results (at 2 h) relative to the engineered CS hydrogel matrices (CS + CS-GBMP1α 1:1) with the different enrichments of P-MC (8% P-MC, 16% P-MC, 24% P-MC) and of T-NC (8% T-NC, 16% T-NC, 24% T-NC). The percentage of viability of cultured human osteoblasts is represented in the y-axis. * indicates a *p*-value < 0.05, ** *p*-value < 0.01, **** *p*-value < 0.0001. (**b**) Proliferation test results, at 2 and 7 days, relative to the engineered CS hydrogel matrices (CS + CS-GBMP1α 1:1) with the different enrichments of P-MC (8% P-MC, 16% P-MC, 24% P-MC) and of T-NC (8% T-NC, 16% T-NC, 24% T-NC). The percentage of detected fluorescent cells is represented in the y-axis. * indicates a *p*-value < 0.05, ** *p*-value < 0.01, *** *p*-value < 0.001, **** *p*-value < 0.0001. (**c**) Mineralization test results (at 7 days) relative to the engineered CS hydrogel matrices (CS + CS-GBMP1α 1:1) with the different enrichments of P-MC (8% P-MC, 16% P-MC, 24% P-MC) and of tunicate T-NC (8% T-NC, 16% T-NC, 24% T-NC). The optical density (OD) of detected alizarin is represented in the y-axis. ** indicates a *p*-value < 0.005, *** *p*-value < 0.001, **** *p*-value < 0.0001.

The results of the MTT (3-(4,5-dimethylthiazole-2-yl)-2,5-diphenyl tetrazolium bromide) adhesion assay showed a statistically significant increase in cell adhesion for the hydrogels enriched with 16% and 24% P-MC compared with the control samples. Furthermore, a progressive increase in the cell viability values was observed with the increasing P-MC concentrations. Specifically, compared with the control, an increase in viability of 26% was recorded in samples containing 8% P-MC, of 40% in samples with 16% P-MC, and of nearly 73% in samples with 24% P-MC (Figure 5a). On the other hand, a significant increase in cell adhesion was observed in hydrogel matrices enriched with 16% and 24% T-NC compared with those containing only chitosan or those enriched with 8% T-NC. Specifically, compared with their control, an increase in viability of 31% was observed in samples containing 16% T-NC and of 37% in samples with 24% T-NC (Figure 5a). This outcome might be attributed to the contribution of cellulose to the biological and mechanical properties of the hydrogel, which may have had an effect on osteoblast behavior [34].

##### Proliferation Test

Matrices enriched with 16% and 24% P-MC demonstrated a statistically significant higher increase in proliferation at 2 days compared with their control sample and the sample containing 8% P-MC. The differences became more pronounced at 7 days, when statistically significant differences were observed among the control hydrogel and all three P-MC concentrations: all the enrichments showed improvements in cell proliferation. Specifically, the percentage of positive cells increased four-fold with the 8% P-MC, six-fold with the 16% P-MC, and over ten-fold with the 24% P-MC concentration. Once again, the 24% P-MC concentration proved to be the most effective (Figure 5b). Proliferation assays on hydrogel matrices enriched with T-NC did not show substantial differences among the three T-NC enrichments after 2 days, as they showed very similar percentages of positive cell numbers (the values were nearly three times higher for 8 and 16% T-NC enrichments and about four times higher for 24%). In contrast, the control sample exhibited slightly lower values. After 7 days, the data revealed that the enrichment with 8% T-NC was approximately 10 times higher than the control hydrogel matrix containing only chitosan, the enrichment with 16% T-NC was nearly 12 times higher, and the last enrichment with 24% T-NC was approximately 18 times higher. Thus, hydrogel matrices enriched with 24% T-NC demonstrated the highest efficiency over time compared with the other samples, particularly at the 7-day mark (Figure 5b). As a result, the combination of pure CS, GBMP1α-functionalized CS, and 24% cellulose proved particularly effective in promoting long-term cell proliferation, positively influencing cell viability. It could be hypothesized that the higher cellulose content might enhance the hydrogel matrix structure, providing three-dimensional mechanical and structural support, which led to greater long-term cell proliferation. The progressive enrichment with cellulose, both P-MC and T-NC, increases the osteoblast proliferation as a consequence of the increase in mechanical properties. The stiffness of the hydrogel can modulate cell behavior and generally leads to an increase in the cell proliferation, mobility, and mineralization of osteoblasts [35]. However, if compared with the enrichment with P-MC, the greatest proliferation was achieved in the hydrogel matrices enriched with T-NC. The different sizes of the T-NC and the P-MC fibers, shown in the TEM images, are reflected in the different morphologies of the hybrid scaffolds. The scaffolds with T-NC appear more homogeneous among themselves than those prepared with P-MC. In particular, the structures appear more porous and show a greater roughness due to both the combination of the fibers and the emergence on their surface of spheres that are similar to those observed in the control of pure chitosan. The better results in the proliferation and mineralization assays of the matrices enriched with tunicate cellulose compared with P-MC could be driven by a greater surface roughness and greater surface area capable of mediating the adsorption of growth factors and proteins.

##### Mineralization Test

The mineralization test results indicated a statistically significant increase for samples enriched with 16% and 24% P-MC compared with their control and with samples enriched with 8% P-MC. A statistically significant difference was also observed between the hydrogel matrices containing 16% P-MC and 24% P-MC, confirming that the higher P-MC concentration showed the best results. Nevertheless, all the tested concentrations showed improvements compared with the control: the matrices enriched with 8% P-MC exhibited an increase of nearly 11% in calcium levels, those enriched with 16% P-MC showed a 37% increase, and those with a 24% P-MC concentration resulted in about a 76% increase (Figure 5c). Furthermore, the hydrogels enriched with T-NC showed positive results in the mineralization test: all of them showed significant increases compared with their control. In particular, compared with the control, an increase in mineralization of 39% was recorded in the sample containing 8% P-MC, 65% in the sample with 16% P-MC, and about 69% in the sample containing 24% P-MC (Figure 5c). The mineralization improves according to the stiffness increase but, as just underlined for the proliferation results, the data indicate a better performance of T-NC samples at 8% and 16% compared with P-MC samples, which is probably due to the different structural features.

## 3. Conclusions

The research interest in sustainable and eco-friendly materials, such as cellulose, has increased dramatically due to their recyclability, biodegradability, compatibility, and non-toxic behavior. In this paper, two celluloses of different origins were blended as structuring agents with a bioactive peptide-engineered chitosan hydrogel. Our results confirm that the viability, proliferation, and calcium deposition of human osteoblasts increased according to the stiffness that was similarly induced by increasing the cellulose concentrations. While the mechanical properties of hybrid matrices vary with the cellulose content but not with the source, the biological properties are different. In the viability at 2 h, the greatest increase (10 times) compared with the control is obtained with 24% P-MC while, at longer times, the most significant results belong to the 24% T-NC matrix with an increase in proliferation at 7 days of 18 times compared with the control. The matrices enriched with T-NC also produce better results in the mineralization assays compared with the matrices with P-MC at the same concentration. The most plausible hypothesis to explain the better biological properties of the matrices enriched with T-NC is the different morphology of the matrices highlighted in the SEM analysis, which demonstrates a different roughness and a greater porosity and homogeneity compared with the matrices containing P-MC. A greater roughness is related to a greater surface area, which in turn influences the adsorption of proteins and growth factors. These characteristics also descend from the different structures between T-NC and P-MC highlighted in the TEM analysis.

Further characterization of these hybrid hydrogels, including dynamic mechanical tests, could complete the comparison between scaffolds with cellulose of different sources, whereas the biological evaluation of the hybrid scaffolds will include the in vivo validation.

## 4. Materials and Methods

### 4.1. Materials

Acetic acid (AcOH), sodium hydroxide (NaOH), acetone, methanol (MeOH), triethylsilane (TES), N,N-dimethylformamide (DMF), dichloromethane (DCM), N,N-diisopropylethylamine (DIPEA), sodium (meta)periodate (NaIO_4_), and acetonitrile were purchased from Sigma-Aldrich (Merck KGaA, Darmstadt, Germany). 2-(1H-benzotriazol-1-il)-1,1,3,3-tetramethyluronium exafluorophosphate (HBTU), ethyl cyano(hydroxyimino)acetate (Oxyma Pure), Rink Amide MBHA resin, sodium cyanoborohydride (NaBH_3_CN), [Ethyl cyano(hydroxyimino)acetato-*O*^2^]tri-1-pyrrolidinylphosphonium hexafluorophosphate (PyOxim), sodium chloride, potassium dihydrogen phosphate, Fmoc-7-aminoheptanoic acid, and all the Fmoc-protected amino acids were purchased from Merck (Darmstadt, Germany). Trifluoroacetic acid (TFA), piperidine, and ethyl ether were from Biosolve (Leenderweg, Valkenswaard, The Netherlands). N-methyl2-pyrrolidone (NMP) was from Iris Biotech GmbH (Marktredwitz, Germania). Ethanol (EtOH) was provided by Carlo Erba (Milan, Italy). CS 70/1000 was purchased from Heppe Medical CS GmbH (Halle, Germany). Potassium chloride and bibasic sodium phosphate were from J.T. Baker Chemicals B.V. (Deventer, The Netherlands). Exilva^®^ P 01-V microfibrillated cellulose was provided by Borregaard (Sarpsborg, Norway).

### 4.2. Methods

#### 4.2.1. Peptide Synthesis

The x-GBMP1α aldehyde peptide (sequence OHC-CO-x-Pro-Phe-Pro-Leu-Ala-Asp-His-Leu-Asn-Ser-Thr-Asn-His-Ala-Ile-Val-Gln-Thr-Leu-Val-Asn-Ser-NH_2_, in which x is the spacer, 7-aminoheptanoic acid) was obtained through the oxidation of the N-terminal serine of the S-x-GBMP1α peptide (sequence H-Ser-x-Pro-Phe-Pro-Leu-Ala-Asp-His-Leu-Asn-Ser-Thr-Asn-His-Ala-Ile-Val-Gln-Thr-Leu-Val-Asn-Ser-NH_2_) with sodium metaperiodate. The S-x-GBMP1α peptide was synthesized with Fmoc chemistry using a solid-phase strategy (Syro I synthetizer, MultiSynTech, Witten, Germany). The solid support was Rink Amide MBHA (substitution 0.62 mmol/g) and the synthesis scale was 0.125 mmol. To ensure proper loading, a double coupling reaction was carried out. The peptide was cleaved from the resin using a mixture of TFA:H_2_O:TES (95:2.5:2.5) for 1.5 h with continuous magnetic stirring. The N-terminal serine was oxidized with 2.5 Mm sodium periodate (NaIO_4_) for 4 min at room temperature, resulting in the peptide with an N-terminal aldehyde group. The successive purification via reverse-phase high-performance liquid chromatography (RP-HPLC, Atlantis dC18 OBD preparative column (10 μm, 100 Å, 10 × 250 mm), Waters, Milford, MA, USA), allowed us to achieve a final product purified to 95% homogeneity [7]. The identity of the final product was confirmed using mass spectrometry: theoretical mass = 2570.943 Da, experimental mass = 2570.77 Da.

#### 4.2.2. CS Functionalization

The first step consists of the preparation of a 40 mL aqueous solution of the x-GBMP1α aldehyde peptide (1 mg/1 mL). CS (40 mg) was dissolved in 2565 µL of 0.2 M AcOH. EtOH (1489 µL) was added to the CS solution and the pH was adjusted to 5.1 with NaOH 0.1 N. The peptide solution was joined to the second one under magnetic stirring, and subsequently, 40 mg of NaBH_3_CN (the same peptide mass) were added and magnetically stirred for 24 h, in order to reduce the Schiff base. Next, the pH was adjusted to 7 with 0.1 N and 1 N NaOH. Afterwards, the same volume of cold EtOH (40.2 mL) was added and stirred for 10 min. Finally, the mixture was left at 4 °C overnight, then filtered with a Gooch 3 filter and dried 1 h under vacuum. In total, 67 mg of functionalized CS were collected.

#### 4.2.3. Cellulose

The plant-based cellulose used for this experiment is the commercial P-MC Exilva^®^ P 01-V. An optical microscopy analysis of this type of fiber, published in a recent study, showed a micrometer length and a diameter of approximately 22–50 µm [36]. It is delivered as a 2% suspension in water, which was lyophilized and used dried for this application. T-NC is extracted from *Phallusia mammillata*; the animals were retrieved from the North Adriatic Sea (in front of Sottomarina, Italy). Their tunic was separated from the internal organs by making an incision starting from the basal part and reaching the oral siphon. Then, all the tunics were rinsed and soaked in water for 5 days, changing water every 24 h. Afterwards, they were carefully washed one by one, attempting to remove most of the algae and encrustations on the surface. Finally, they were cut into pieces with approximate lateral dimensions of 1 cm × 1 cm and immersed in a 5 wt% H_2_SO_4_ solution for 72 h in constant magnetic stirring. Subsequently, the tunic pieces were separated from the solution using a 2 × 2 mm mesh strainer and placed in a stirrer with 9% (*v*/*v*) NaOH solution. After 3 h, the fragments were isolated again and rinsed with deionized water until a pH of 7 was reached. The extracted hydrogel pieces were then subjected to a homogenization process using Ultraturrax in order to prepare a homogeneous nanocellulose dispersion. The suspension was dried in an oven at 45 °C under vacuum [37].

#### 4.2.4. Preparation of Cellulose-Enriched Matrices

**Figure 6:**
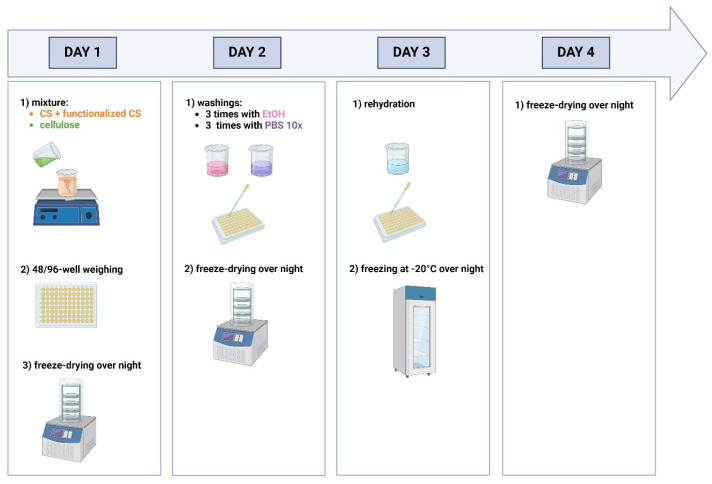
Schematic protocol for the realization of functionalized CS- and cellulose-based matrices.

To evaluate the structural properties of P-MC and T-NC, different matrices were tested. The control matrix (CTRL) was prepared with 50% CS and 50% CS functionalized with peptide GBMP1α. In particular, CTRL1 and CTRL2 are the control samples, respectively made up of CS1 and CS2, i.e., of CS-GBMP1α used to obtain matrices enriched with cellulose of vegetal or tunicate origin, respectively. According to our previous studies, this matrix represents the best condition to optimize human osteoblast adhesion, growth, and proliferation, while maintaining good mechanical properties and without reducing the antimicrobial properties of CS [11]. Two different sizes of hydrogel matrices were realized: for the smallest one, 200 mg of the final mixture were weighed in 96-well plates, while for the biggest one, 500 mg of the final mixture were weighed in 48-well plates. For a single control matrix of the smallest size, CS (0.84 mg) and functionalized CS (0.84 mg) were dissolved in 242 μL of 0.2 M AcOH. The other matrices were prepared with the enrichment of P-MC (8% *w*/*w*, 16% *w*/*w,* and 24% *w*/*w*) or with the enrichment of T-NC (8% *w*/*w*, 16% *w*/*w,* and 24% *w*/*w*) The cellulose enrichments were decided starting from the indication reported in [38]. To prepare a single cellulose-enriched hydrogel matrix of the smallest size, CS (0.42 mg) and functionalized CS (0.42 mg) were dissolved in 121 μL of 0.2 M AcOH and then mixed with the various specific amounts of cellulose suspended in a fixed volume of 266 μL of MilliQ water: in particular, 0.0672 mg of cellulose were used for the matrix with 8% *w*/*w* enrichment, 0.1344 mg for the matrix with 16% *w*/*w* enrichment, and 0.2016 mg for the matrix with 24% *w*/*w* enrichment. To create a single control or cellulose-enriched matrix of the biggest size, the procedure was the same as described above but with doubled amounts. Subsequently, the matrices were frozen with liquid nitrogen, freeze-dried, and then washed in an ultrasound bath 3 times with EtOH and 3 times with phosphate-buffered saline 10× (PBS, made up of sodium chloride 1.37 M, potassium chloride 27 mM, bibasic sodium phosphate 0.1 M, potassium dihydrogen phosphate 18 mM), and finally freeze-dried. The matrices were re-hydrated with MilliQ water and left at −20 °C overnight and lyophilized. The protocol is reported in the scheme (Figure 6).

### 4.3. Chemical Characterization of Peptide-Grafted CS

#### NMR Experiment

NMR analyses were performed on CS and CS functionalized with the GBMP1α peptide (CS-GBMP1α). The NMR sample of CS, useful for estimating the degree of acetylation of the polymer, was obtained by dissolving ~0.8 mg of chitosan in 0.700 mL of D_2_O (isotopic purity 99.9%) containing ~1% (*v*:*v*) of 12 M HCl aqueous solution (Sigma-Aldrich product). Given the quantity of CS-GBMP1α necessary for the preparation of our matrices, pools of the adduct were prepared at different times, starting from the same source of CS. Each preparation was NMR-characterized in order to evaluate the degree of functionalization of CS by the peptide. We named CS1 as the CS-GBMP1α adduct used to obtain the matrices enriched with cellulose of plant origin, and CS2 as the CS-GBMP1α used to obtain the matrices enriched with tunicate cellulose. NMR samples of CS1 and CS2 were typically prepared by dissolving 0.7–0.8 mg of the product in 0.700 mL of deuterated acetic acid (isotopic purity ≥ 99.9%) 2M in D_2_O (isotopic purity 99.9%). Acetic acid-d_4_ and D_2_O were purchased from Merck Life Science Srl, Milan, Italy. Of note, the concentrations of CS1 or CS2 cited above were necessary to avoid viscous solutions and wide linewidths of the NMR signals. NMR spectra were performed at 298 and 313 K using Bruker Avance spectrometers operating at 600 and 700 MHz ^1^H Larmor frequencies. The proton spectra were typically acquired with 32 scans, a 30 s interscan delay, and water suppression with z-gradients. Trimethylsilyl propanoic acid (TSP, Aldrich) at 0.0 ppm was used for the internal reference of the spectra.

### 4.4. Morphological Characterization of Cellulose

#### TEM

To perform the TEM analysis, 25 μL of P-MC suspension and 25 μL of T-NC suspension were used. These were deposited onto a piece of parafilm to form droplets. A copper grid, which has 37 μm-wide holes, was placed on the samples, which were then stained with 1% uranyl acetate for 2 min and observed using a FEI Tecnai G2 transmission electron microscope at 100 kV. Images were captured using a Veleta digital camera (Olympus Soft Imaging System). An image-recognition tool (ImageJ, National Institutes of Health, Madison, USA) was utilized to investigate the morphology of cellulose fibers. The average width, length, and aspect ratio were obtained considering 10 measures.

### 4.5. Characterization of Hybrid Scaffolds

#### 4.5.1. Morphological Characterization

##### SEM

Freeze-dried hydrogel matrices were sputtered with gold (EMITECHK950x Turbo Evaporator, EBSciences, East Granby, CT, USA) and observed under scanning electron microscopy, SEM (Cambridge Stereoscan 440 SEM, Cambridge, UK). The images were recorded at 100×, 300×, and 600× magnifications using an accelerating voltage of 20 kV.

#### 4.5.2. Mechanical Measurements

##### Compression Tests

Compression tests were carried out on several cylindrical scaffolds with an initial radius R_0_ of 5.0 mm and a height of 0.3 mm. The specimens were immersed in saline solution at 37 °C and compressed using an INSTRON 5566 testing machine at a speed of 1 mm/min to a strain level of 0.4 mm/mm. Based on the measured force (*F*), the initial area of the specimen (*A*_0_ = πR_0_^2^), the change in the specimen height (Δ*h*), the initial height of the specimen (*h*_0_), the engineering stress (*σ*), and the engineering strain (*ε*) were calculated as reported below:

(1) $$\sigma =\frac{F}{A_{0}}$$

(2) $$\varepsilon =\frac{∆h}{h_{0}}$$

#### 4.5.3. Biological Characterization

##### Human Osteoblast Cell Isolation and Culture

The hydrogel matrices were biologically assessed using primary human osteoblasts isolated from the mandibular bone of healthy subjects undergoing a surgical procedure. The study was approved by the Ethical Committee of the University Hospital of Padova (Aut. 4899/AO/20 on May 5, 2020). Human osteoblasts were cultivated at 37 °C in an atmosphere of 5% CO_2_ in cell culture flasks containing a complete medium composed of DMEM (Dulbecco’s Modified Eagle Medium, ThermoFisher Sci, Milan, Italy) enriched with 10% (*v*/*v*) heat-inactivated fetal bovine serum (FBS), 10 U/mL of streptomycin and penicillin, 1% (*v*/*v*) non-essential amino acids, 1% (*v*/*v*) sodium pyruvate, and 1 U/mL of insulin, all provided by ThermoFisher Sci. Upon reaching the confluence, the osteoblasts were detached through incubation with trypsin–EDTA and incubated in a complete medium added with 5 μg/mL of ascorbic acid, 10 nM of dexamethasone, and 10 mM of β-glycerophosphate to induce cellular differentiation. After an additional 6 days of culture, the cells were detached using trypsin–EDTA, seeded on matrices, and incubated at 37 °C.

##### Viability Assay

The metabolic activity of the cells was evaluated using the MTT assay. For the MTT test, cells (3 × 10^5^/cm^2^ in 200 μL DMEM) were cultured at 37 °C for 2 h on matrices placed in ultra-low attachment 96-well plates (Corning^®^ Costar^®^; Sigma). Cells were rinsed twice with PBS to remove non-adherent cells and then incubated with 200 μL of MTT (Merck; Milan, Italy) solution (5 mg/mL in PBS) at 37 °C for 2 h. The reaction was stopped by adding 0.01 N HCl in 10% *w*/*v* SDS. Since the MTT assay evaluates the metabolic cell activity, we inferred the number of cells adhering to the hydrogel matrices by setting a standard curve obtained for each experiment and seeding a known number of cells in tissue culture plates. At the end of the incubation, cellular lysates were transferred in 96-well plates to determine the absorbance at 620 nm using a microplate reader (MultiPlateReader VictorX2 (Perkin Elmer, Milan, Italy)). The cell survival ratio was expressed as a percentage with respect to the cells seeded on the plastic surface; the values obtained from the control samples were set equal to 100%. Data were obtained from two replicates of three independent experiments.

##### Proliferation Assay

Human osteoblast cells were initially incubated at 37 °C for 10 min with 25 μM of carboxyfluorescein diacetate succinimidyl ester (CFSE), a fluorescent probe that is capable of permeating into cells, which was evenly distributed among the daughter cells during the cell division process. This reaction was stopped by the addition of 5 volumes of cold PBS and subsequent centrifugation. Osteoblasts were then seeded and incubated on the matrices at 37 °C for 2 or 7 days. At the end of the incubation, the cells were detached by treatment with trypsin, washed, and centrifuged. The distribution of fluorescence among the daughter cells was indicative of cell proliferation and was evaluated using a FACS (fluorescence-activated cell sorting) analysis, using a BD FACSCalibur. For each analysis, 10,000 events were collected.

##### Mineralization Assay

The Alizarin Red S staining assay was used to assess calcium deposition. Cells were cultured on the matrices for 7 days at 37 °C. Cultures were then washed twice with PBS and fixed with 4% *vol*/*vol* para-formaldehyde (PFA) for 10 min at room temperature. Additional PBS washings were performed, after which the samples were incubated with a 40 mM Alizarin Red S solution (Merck) at room temperature for 30 min under agitation. Therefore, the samples were washed and incubated at −20 °C for 30 min, followed by the addition of a 10% *v*/*v* AcOH solution, and then incubated for 30 min at room temperature under agitation. Then, the osteoblasts were vortexed for 30 s and incubated at 85 °C for 10 min. The lysis reaction was stopped by placing the samples on ice for 5 min. After centrifugation at 20,000 rpm for 15 min, the absorbance of the supernatant was recorded at 450 nm using a microplate reader (MultiPlateReader VictorX2, Perkin Elmer, Waltham, MA, USA). A standard curve with known concentrations of Alizarin Red S and CaCo_3_ was simultaneously generated and used to calculate the concentration of Alizarin and determine the amount of calcium in each hydrogel sample. All the collected data were normalized to their respective controls to evaluate the contribution of cellulose.

##### Statistical Analysis

Data obtained from the biological assays were analyzed and processed using Prism (GraphPad Software v.9). The results of the MTT viability test and the Alizarin Red S mineralization assay were compared using the one-way analysis of variance test (one-way ANOVA). The results of the proliferation assay at two different timepoints were compared using the two-way analysis of variance test (two-way ANOVA). The Tukey’s multiple comparison test was chosen to compare all the conditions. The level of significance applied was 5%.

## Figures and Tables

**Table 1:** The width and length of cellulose fibers, reported as mean value ± standard deviation, were measured using ImageJ software (Version 1.51). The aspect ratio was calculated as the ratio between the length and width of the fibers.

Sample	Width (nm)	Length (nm)	Aspect Ratio
P-MC	15.500 ± 2.824	215.638 ± 86.646	13.9
T-NC	5.847 ± 1.166	1526.427 ± 433.805	261.1

**Table 2:** Results of the mechanical measurements: compressive modulus (E) and maximum stress (σ_max_) for the control groups (CTRL1, CTRL2) and all the hydrogel matrices enriched with P-MC and with T-NC. The results are reported as mean value ± standard deviation.

	CTRL1 (CS + CS1 1:1)	+8% P-MC	+16% P-MC	+24% P-MC	CTRL2 (CS + CS2 1:1)	+8% T-NC	+16% T-NC	+24% T-NC
σ_max_ [MPa]	0.15 ± 0.01	0.20 ± 0.01	0.23 ± 0.01	0.28 ± 0.02	0.15 ± 0.01	0.19 ± 0.01	0.22 ± 0.02	0.28 ± 0.02
E [MPa]	0.4 ± 0.1	0.8 ± 0.2	1.0 ± 0.2	1.4 ± 0.2	0.5 ± 0.1	0.7 ± 0.2	0.9 ± 0.2	1.3 ± 0.3

## Data Availability

The raw data supporting the conclusions of this article will be made available by the authors on request.

## Supplementary Materials

The following supporting information can be downloaded at: https://www.mdpi.com/article/10.3390/gels11020102/s1, Figure S1: NMR spectra. Figure S2: Results from mechanical measurements.
