# Supplementary material for: Comparative Analysis of Tunicate vs. Plant-Based Cellulose in Chitosan Hydrogels for Bone Regeneration
Source: Gels. 2025 Feb 1;11(2):102. doi: 10.3390/gels11020102 (PMC11854000; doi:10.3390/gels11020102)
Supplement: Supplementary file 1 [file gels-11-00102-s001.zip › gels-3372161-supplementary.pdf]

Supplementary Materials

Comparative Analysis of Tunicate vs. Plant-Based Cellulose in Chitosan Hydrogels for Bone Regeneration

Laura Furlan [1], Annj Zamuner [1,2], Andrea Riccioni [3], Giacomo Sabbadin [4], Teresa Russo [5], Vito Gallicchio [6], Gabriella D'Auria [7], Lucia Falcigno [7], Lucia Manni [4], Loriano Ballarin [4], Elisabetta Schievano [3], Paola Brun [8] and Monica Dettin [1,*]

[1] Department of Industrial Engineering, University of Padova, 35131 Padova, Italy; laura.furlan@unipd.it (L.F.); annj.zamuner@unipd.it (A.Z.)
[2] Department of Civil, Architectural and Environmental Engineering, University of Padova, 35131 Padova, Italy
[3] Department of Chemical Sciences, University of Padova, 35131 Padova, Italy; andrea.riccioni@polito.it (A.R.); elisabetta.schievano@unipd.it (E.S.)
[4] Department of Biology, University of Padova, 35131 Padova, Italy; giacomo.sabbadin.1@studenti.unipd.it (G.S.); lucia.manni@unipd.it (L.M.); loriano.ballarin@unipd.it (L.B.)
[5] Institute of Polymers, Composites and Biomaterials, National Research Council of Italy, 80125 Naples, Italy; teresa.russo@unina.it
[6] Department of Neurosciences, Reproductive and Odontostomatological Sciences, University of Naples Federico II, 80131 Naples, Italy; vito.gallicchio@unina.it
[7] Department of Pharmacy, University Federico II of Naples, 80131 Naples, Italy; gabriella.dauria@unina.it (G.D.); falcigno@unina.it (L.F.)
[8] Department of Molecular Medicine, University of Padova, 35121 Padua, Italy; paola.brun.1@unipd.it
* Correspondence: monica.dettin@unipd.it; Tel.: +39-049-827-5553

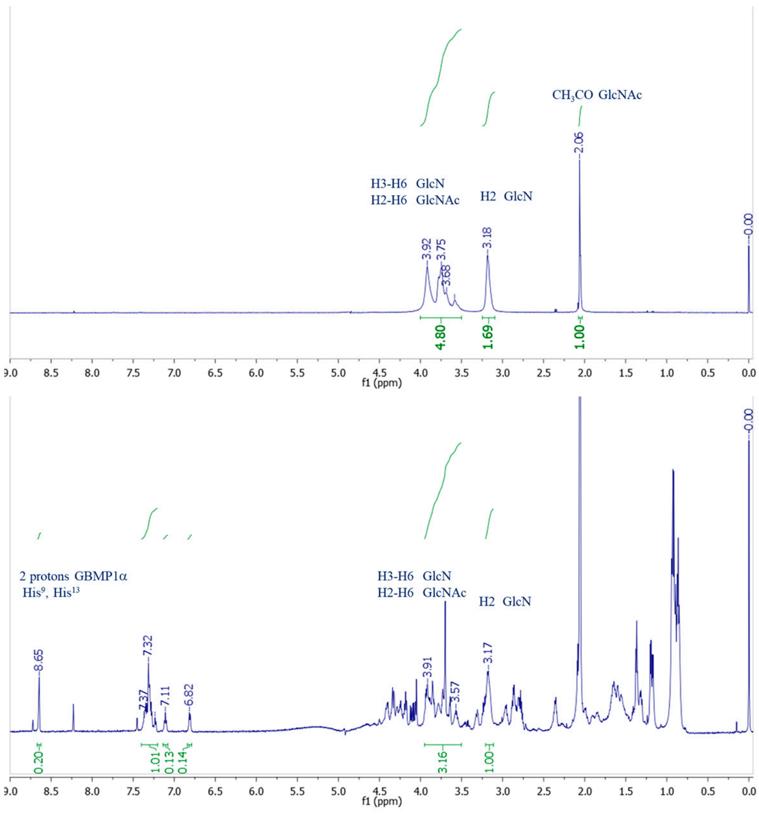

(**a**)

(**b**)

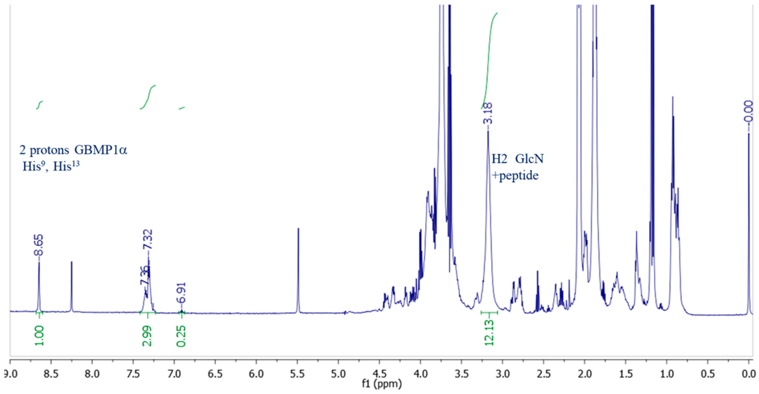

(**c**)

Figure S1. NMR spectra. (a) ^{1}H spectrum of CS in acidic D_2O (2% HCl) acquired at 298 K and 600 MHz. Chemical shift (ppm) and integral values are reported in blue and green above and below the signals, respectively. (b) ^{1}H spectra of CS1 in perdeuterated acetic acid 2M in D_2O acquired at 298 K and 700 MHz. Chemical shift (ppm) and integral values are reported in blue and green above and below the signals. respectively. (c) ^{1}H spectra of CS2 in perdeuterated acetic acid 2M in D_2O acquired at 298 K and 600 MHz. Chemical shift (ppm) and integral values are reported in blue and green above and below the signals, respectively.

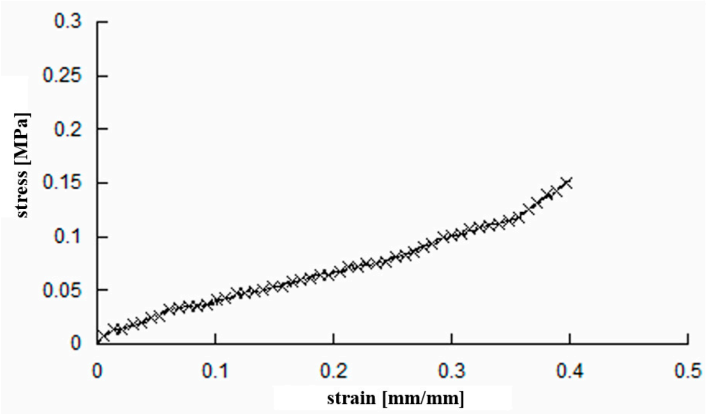

(**a**)

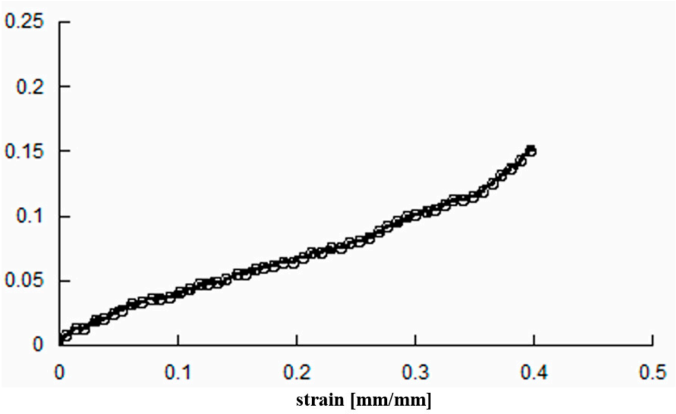

(**b**)

Figure S2. Results from mechanical measurements. (a) A typical stress-strain curve for peptide-grafted chitosan matrices enriched with (a) P-MC and (b) T-NC and their relative control samples.
